# Low birth weight trends in Organisation for Economic Co-operation and Development countries, 2000–2015: economic, health system and demographic conditionings

**DOI:** 10.1186/s12884-020-03484-9

**Published:** 2021-01-06

**Authors:** Diego Erasun, Jéssica Alonso-Molero, Inés Gómez-Acebo, Trinidad Dierssen-Sotos, Javier Llorca, José Schneider

**Affiliations:** 1 University Hospital Marqués de Valdecilla, Santander, Spain; 2 Department of Preventive Medicine and Public Health, University of Cantabria, Avda. Herrera Oria s/n, 39011 Santander, Spain; 3 IDIVAL, Santander, Spain; 4 CIBER Epidemiología y Salud Pública (CIBERESP), Madrid, Spain; 5 Universidad de Valladolid, Valladolid, Spain

**Keywords:** Low birth weight, Developed countries, Health system, Pediatricians, Delivery, Obstetric, Caesarean section

## Abstract

**Background:**

Low birth weight rates are increasing in both developed and developing countries. Although several maternal factors have been identified as associated with low birth weight, little is known of economic or organization factors influencing this increase. This study aims to ascertain the twenty-first century relationships between the contextual country factors and low birth weight rates.

**Methods:**

We analyse trends of low birth weight rates in Organisation for Economic Co-operation and Development (OECD) countries. Data from 2000 to 2015 were obtained from the OECD data base. Their relationships with demographic and economic variables, health habits, woman-related preventive measures, health care system organization and funding, health care work force and obstetric care were analysed using random-effects linear regression.

**Results:**

Low birth weight rates are higher in Southern Europe (7.61%) and lower in Northern Europe (4.68%). Low birth weight rates escalated about 20% in Southern Europe and to less extent in Easter Europe (7%) and Asian/Oceanian countries, while remained stable in America, Central Europe and Northern Europe. Investment in health care, private health system coverage, ratios of paediatricians and obstetricians, average length of admission due to pregnancy or birth and Caesarean section rate were associated with higher low birth weight rates. Factors associated with lower low birth weight rates were health care coverage, public health system coverage, hospitals per million inhabitants, and ratios of health care workers, physicians, midwives and nurses.

**Conclusions:**

In OECD countries, LBW rates are related to contextual country characteristics such as GDP per capita, which is inversely related to LBW rate. Health care system factors, including health care coverage or investment in public health system, are directly associated with lower LBW rates.

**Supplementary Information:**

The online version contains supplementary material available at 10.1186/s12884-020-03484-9.

## Background

Low birth weight (LBW) is defined as birth weight ≤ 2500 g by the World Health Organization, not considering gestational age [1]. LBW is a key factor because it affects about 15.5% of all newborns [2]. LBW babies are at higher risk of neonatal morbidity and mortality [2], including higher risk for developing diseases such as childhood obesity, hypertension, adverse cardiovascular and metabolic outcomes or impaired neurodevelopment [2, 3]. LBW is associated with enhanced infant mortality and long-term morbidity during adulthood [4], which makes weight at birth a relevant indicator for the fetus’ development quality and a predictor of health throughout its life course [5].

LBW is associated not only with maternal factors or pathological causes but also with contextual country factors. Maternal factors associated with LBW are well known. They include factors such as maternal age [6–8], race [9], high pre-gestational maternal weight or a great weight gain during pregnancy [10–12], smoking and alcohol consumption during pregnancy [13, 14], gestational diabetes, type of diet, as plant-based diet [15, 16], low socio-economic status or low maternal education [2, 17–19]. Regarding pathological causes, prematurity and intrauterine growth retardation are the most prominent ones [20, 21].

Little is known, however, on the relationship between LBW and contextual (i.e. general) country factors. Factors as, for instance, the evolution of per capita Gross Domestic Product (GDP), budget dedicated to the health system, number of physicians, nurses, or other health care professionals, or health system performance have been little studied. Most of recent studies analyze the impact of the global financial crisis of 2008 on LBW [17, 22–24], confirming the significant increase in LBW during that crisis. Countries where the most severe austerity measures were implemented have experienced the highest increase in LBW rate [17, 22–24].

The Organisation for Economic Co-operation and Development (OECD) conducts annually an evaluation of health variables entitled “Heath at a glance”, presenting an analysis with aggregated data on different health variables. “Health at a glance” dedicates a specific chapter to children’s health, where it collects data on LBW [25].

The purpose of this study was to ascertain the twenty-first century relationships between the contextual country factors and low birth weight rates in OECD countries.

## Methods

### Source of information and data gathered

We gathered data for all 35 OECD countries (Supplementary Table 1) for the period ranging from 2000 to 2016 from the OECD data base (https://data.oecd.org/; last access: 19/October/2018). Data for 2016 could not be used in this analysis as most information on LBW was still not available at the date the data base was consulted. For descriptive purposes, countries were classified into six regions (America, Eastern Europe [countries in the former Eastern block], Northern Europe [Scandinavian countries, UK and Ireland], Southern Europe [European countries with Mediterranean coast], Central Europe [all other European countries] and other countries [Asian and Oceanian countries]) (Supplementary Table 1).

Information obtained included LBW rate (per 100 newborns), demographic and economic variables (percentage of women in the general population, fertility and natality rates, per capita GDP), health habits and woman-related preventive measures (smoking, ethanol consumption, people reporting good health, breast and cervical cancer screening coverages), health care system organization and funding (investment in health care, health care coverage, public and private system coverage, number of hospitals), health care work force (health care workers, physicians, general practitioners, paediatricians, obstetricians, midwives, nurses) and obstetric care (average length of admission due to pregnancy, single birth, other births and puerperium-associated complications, and Caesarean rate).

### Statistical analysis

Data were arranged as panels according to country and year. Relationships between explainable variables and LBW rates were analysed using random-effects linear regression. Random-effects models were used under the assumption that trends in LBW rates have two sources of variability, one related with the year and another related with the country; therefore, they would account for the existence of between-country different trends. Results are presented as linear regression coefficient with 95% confidence interval (CI). All analyses were carried out with the Stata 16/SE statistical package (Stata Corp., College Station, Texas, US).

## Results

Mean and standard deviations for all variables recorded are displayed in Table 1, stratified by 5-year periods. LBW average rate increased from 6.2% in 2005 to 6.5% in 2015. Fertility and natality rate showed inconsistent trends, while per capita GDP about doubled its average from 2000 to 2010 but slightly decreased towards 2015. Average number of cigarettes and ethanol consumption declined throughout the period, whereas the percentage of people reporting good health and breast cancer screening coverage increased. Investment in health care escalated from 7.2% GDP to 8.9% GDP, but trends in health care coverage were inconsistent. Physician ratios increased all over the studied period as well as ratios of GPs, paediatricians, obstetricians and nurses. Average length of admission due to obstetrics procedures decreased, but Caesarean section rates escalated from 181 to 252 per 1000 newborns.

**Table 1:** Description of the variables included in this study: mean ± standard deviation in 2000, 2005, 2010 and 2015 in OECD countries

Variable	2000	2005	2010	2015
**Low weight at birth (per 100 new-borns)**	6.2 ± 1.5	6.4 ± 1.5	6.5 ± 1.6	6.5 ± 1.6
**Demographic and economic characteristics**				
**Women in general population (percentage)**	51.1 ± 0.9	51.0 ± 0.9	51.0 ± 1.0	50.9 ± 1.0
**Fertility rate (per woman in fertility age)**	1.67 ± 0.41	1.65 ± 0.38	1.74 ± 0.37	1.68 ± 0.35
**Natality rate (per 1000 inhabitants)**	12.38 ± 3.64	11.82 ± 3.01	12.26 ± 3.33	10.89 ± 2.80
**Per capita GDP (thousand $)**	19.8 ± 12.3	31.2 ± 18.4	37.8 ± 22.0	36.8 ± 21.5
**General health, healthy habits and preventive measures**				
**Number of cigarettes per smoker and day**	14.6 ± 2.9	14.4 ± 2.1	13.7 ± 2.1	12.0 ± 2.2
**Ethanol (yearly litres per capita)**	9.4 ± 3.1	9.7 ± 2.9	9.2 ± 2.4	8.7 ± 2.5
**People aged 15–24 reporting good health (percentage)**	82.1 ± 9.9	89.4 ± 7.8	89.3 ± 11.5	89.5 ± 9.3
**People aged 25–44 reporting good health (percentage)**	74.4 ± 13.7	80.1 ± 11.2	81.8 ± 13.0	82.1 ± 11.2
**Breast cancer screening (coverage in percentage)**	47.0 ± 30.8	53.7 ± 23.0	59.6 ± 19.0	57.1 ± 18.9
**Cervical cancer screening (coverage in percentage)**	64.5 ± 22.0	55.9 ± 21.4	55.2 ± 18.4	56.9 ± 17.8
**Health care system organization and funding**				
**Investment in health care (percentage of GDP)**	7.2 ± 1.7	8.0 ± 1.9	8.8 ± 2.1	8.9 ± 2.3
**Health care coverage (percentage)**	98.7 ± 3.8	96.8 ± 8.5	97.9 ± 4.4	97.7 ± 3.8
**Public health system coverage (percentage)**	94.3 ± 16.5	93.1 ± 16.4	95.4 ± 13.1	95.1 ± 12.3
**Private health system coverage (percentage)**	5.0 ± 14.2	4.7 ± 13.5	3.1 ± 10.9	3.7 ± 12.1
**Hospitals per million inhabitants**	35.1 ± 20.4	33.3 ± 17.4	30.6 ± 14.2	29.3 ± 14.8
**Health care work force**				
**Health care workers (per 1000 inhabitants)**	41.4 ± 20.6	43.0 ± 22.7	46.0 ± 24.5	48.3 ± 22.6
**Physicians (per 1000 inhabitants)**	2.70 ± 0.65	2.90 ± 0.68	3.11 ± 0.73	3.31 ± 0.71
**General practitioners (per 1000 inhabitants)**	0.93 ± 0.40	0.96 ± 0.55	0.94 ± 0.53	1.06 ± 0.48
**Paediatricians (per 1000 inhabitants)**	0.13 ± 0.07	0.13 ± 0.06	0.14 ± 0.07	0.16 ± 0.08
**Obstetricians (per 1000 inhabitants)**	0.13 ± 0.05	0.13 ± 0.05	0.14 ± 0.05	0.15 ± 0.06
**Obstetricians (per 1000 births)**	11.5 ± 6.3	11.9 ± 6.0	12.0 ± 5.8	15.7 ± 7.5
**Midwifes (per 1000 inhabitants)**	0.34 ± 0.19	0.32 ± 0.21	0.35 ± 0.21	0.38 ± 0.25
**Midwifes (per 1000 births)**	31.5 ± 17.3	29.6 ± 17.1	29.9 ± 14.9	33.7 ± 16.1
**Nurses (no midwifes) (per 1000 inhabitants)**	8.30 ± 3.47	8.98 ± 3.79	10.0 ± 4.35	9.77 ± 4.65
**Obstetrics care**				
**Average length of admission due to pregnancy**	4.1 ± 1.2	3.9 ± 1.1	3.7 ± 1.0	3.5 ± 0.9
**Average length of admission due to single birth**	3.6 ± 1.4	3.4 ± 1.2	3.1 ± 1.1	3.0 ± 1.1
**Average length of admission due to other births**	6.0 ± 2.5	5.6 ± 2.0	4.8 ± 1.5	4.4 ± 1.3
**Average length of admission due to puerperium-associated complications**	4.3 ± 0.9	4.2 ± 1.2	3.9 ± 0.8	3.8 ± 0.6
**Caesarean rate (per 1000 new-borns)**	180.8 ± 49.4	228.9 ± 68.1	265.5 ± 76.0	251.9 ± 72.5

**Fig. 1:**
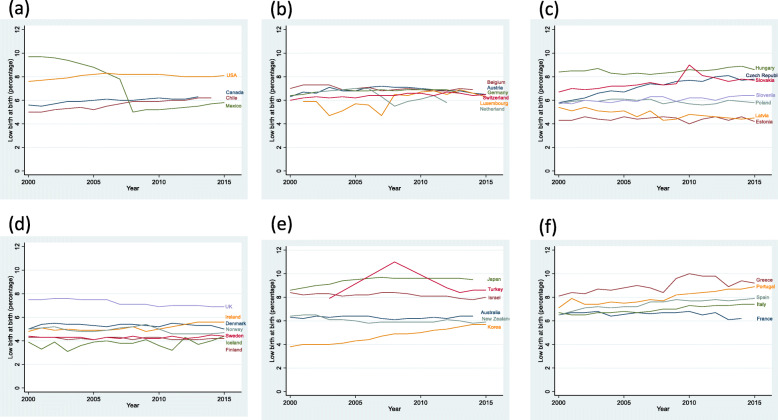
Trends in low weight at birth rates from 2000 to 2015 in OECD countries, by region. **a** America, **b** Central Europe, **c** East Europe, **d** North Europe, **e** Other countries (Asian / Oceanian), **f** South Europe

Considering the whole period, LBW was more frequent in Southern Europe (7.61, 95%: 7.34–7.88) and less frequent in Northern Europe (4.98%, 4.75–5.21) (Table 2). From 2000 to 2015, LBW rates escalated about 20% in Southern Europe, 7% in Eastern Europe, 5% in Asian/Oceanian countries and remained stable in America, Central Europe and Northern Europe. Figure 1 shows LBW rate trends in each country. In American countries, Chile and Canada reported similar figures and trends, while the US had higher rates and Mexico displayed a somewhat erratic trend (Fig. 1a). All seven Central European countries had steady trends and similar rates (Fig. 1b), being the most homogeneous region as shown by the small standard deviations in Table 2. Trends in Eastern Europe were somewhat divergent, with Estonia and Latvia having lower rates and some decreasing trends, and Hungary, Czech Republic and Slovakia having higher rates and raising trends (Fig. 1c). All countries in Northern Europe except the United Kingdom had low rates of LBW; the United Kingdom, however, was the only one displaying a decreasing trend (Fig. 1d). Countries grouped in “Other” category were highly heterogenous; Korea and Japan had the lowest and the highest LBW rates respectively and both exhibit a stepping trend (Fig. 1e). Finally, all five countries in Southern Europe had high LBW rates, France being the only one not showing a raising trend (Fig. 1f).

**Table 2:** Low weight at birth averages by region of OECD countries (2000–2015)

Region	Low weight at birth average rate (95% confidence interval), 2000–2015	Low weight at birth average rate ± standard deviation in 2000	Low weight at birth average rate ± standard deviation in 2005	Low weight at birth average rate ± standard deviation in 2010	Low weight at birth average rate ± standard deviation in 2015	% change from 2005 to 2015
**Southern Europe**	7.61 (7.34–7.88)	6.98 ± 0.67	7.58 ± 0.87	8.02 ± 1.24	8.35 ± 0.84	+ 19.6
**Other countries**	7.04 (6.78–7.31)	6.70 ± 1.95	6.88 ± 2.02	6.96 ± 1.86	7.05 ± 1.47	+ 5.2
**America**	6.75 (6.44–7.06)	6.98 ± 2.13	7.05 ± 1.72	6.38 ± 1.29	6.95 ± 1.70	−0.4
**Central Europe**	6.57 (6.31–6.83)	6.43 ± 0.42	6.58 ± 0.52	6.70 ± 0.33	6.43 ± 0.12	+ 0.1
**Eastern Europe**	6.33 (6.10–6.56)	6.01 ± 1.27	6.27 ± 1.23	6.57 ± 1.91	6.43 ± 1.70	+ 7.0
**Northern Europe**	4.98 (4.75–5.21)	4.99 ± 1.18	4.94 ± 1.24	4.89 ± 1.05	5.03 ± 0.95	+ 0.8

The associations between explainable variables and LBW rates appear in Table 3. Fertility and natality rates were not associated with LBW rate. Per capita GDP was inversely associated with LBW, although a thousand dollar ($1000) increase in “per capita” GDP only accounted for a 0.012% decrease in LBW (95% CI: − 0.018, − 0.007). Countries with higher number of cigarettes per smoker reported higher LBW rates, while countries with higher breast or cervical cancer screening coverages reported lower LBW. In contrast to demographic and economic characteristics or general health, healthy habits and preventive measures, all variables on health care system organization and funding were associated with LBW rates: the higher the investment in health care or the private health system coverage, the higher the LBW rate. Health care coverage, public health system coverage and hospitals per million inhabitants were negatively associated with LBW rates. Ratios of health care workers, physicians, midwives and nurses were negatively associated with LBW rates, while higher ratios of paediatricians and obstetricians were associated with higher LBW rates. Finally, most obstetric care indicators (average length of admission due to pregnancy-related factors, single birth or puerperium-associated complications, as well as the Caesarean rate) were related with higher LBW rates.

**Table 3:** Variables associated with low weight at birth in OECD countries (2000–2015)

Variable	Coefficient (95% CI)	p
**Demographic and economic characteristics**		
**Women in general population (percentage)**	0.15 (0.01, 0.29)	0.03
**Fertility rate (per woman in fertility age)**	−0.17 (−0.50, 0.17)	0.33
**Natality rate (per 1000 inhabitants)**	0.002 (−0.041, 0.045)	0.92
**Per capita GDP (thousand $)**	−0.012 (− 0.018, − 0.007)	< 0.001
**General health, healthy habits and preventive measures**		
**Number of cigarettes per smoker and day**	0.20 (0.13, 0.27)	< 0.001
**Ethanol (yearly litres per capita)**	−0.04 (− 0.09, 0.01)	0.11
**People aged 15–24 reporting good health (percentage)**	0.02 (0.01, 0.04)	0.009
**People aged 25–44 reporting good health (percentage)**	0.007 (−0.006, 0.021)	0.29
**Breast cancer screening (coverage in percentage)**	−0.022 (− 0.029, − 0.014)	< 0.001
**Cervical cancer screening (coverage in percentage)**	− 0.028 (− 0.036, − 0.019)	< 0.001
**Health care system organization and funding**		
**Investment in health care (percentage of GDP)**	0.12 (0.06, 0.18)	< 0.001
**Health care coverage (in percentage)**	−0.041 (− 0.061, − 0.022)	< 0.001
**Public health system coverage (percentage)**	− 0.021 (− 0.030, − 0.012)	< 0.001
**Private health system coverage (percentage)**	0.025 (0.014, 0.035)	< 0.001
**Hospitals per million inhabitants**	−0.023 (− 0.031, − 0.014)	< 0.001
**Health care work force**		
**Health care workers (per 1000 inhabitants)**	− 0.020 (− 0.027, − 0.013)	< 0.001
**Physicians (per 1000 inhabitants)**	−0.205 (− 0.411, 0.000)	0.05
**General practitioners (per 1000 inhabitants)**	0.147 (−0.140, 0.434)	0.32
**Paediatricians (per 1000 inhabitants)**	9.39 (7.52, 11.27)	< 0.001
**Obstetricians (per 1000 inhabitants)**	7.31 (4.63, 9.99)	< 0.001
**Obstetricians (per 1000 births)**	0.031 (0.009, 0.054)	0.006
**Midwifes (per 1000 inhabitants)**	−2.64 (−3.39, −1.90)	< 0.001
**Midwifes (per 1000 births)**	−0.026 (−0.036, − 0.016)	< 0.001
**Nurses (no midwifes) (per 1000 inhabitants)**	−0.170 (− 0.206, − 0.134)	< 0.001
**Obstetrics care**		
**Average length of admission due to pregnancy**	0.243 (0.124, 0.363)	< 0.001
**Average length of admission due to single birth**	0.214 (0.110, 0.319)	< 0.001
**Average length of admission due to other births**	0.016 (−0.053, 0.085)	0.65
**Average length of admission due to puerperium-associated complications**	0.500 (0.352, 0.648)	< 0.001
**Caesarean rate (per 1000 newborns)**	0.0073 (0.0058, 0.0088)	< 0.001

## Discussion

An increase in low birth weight rates has been observed between 2000 and 2015 in some OECD countries, and most particularly the Southern European countries, especially Greece, Portugal, Spain, with the UK at the opposite geographical extreme (Fig. 1). Some authors [17, 22, 23] considered that the increase in LBW rate during the 2008–2014 period cannot be explained only by maternal factors. These authors relate the financial crisis starting in 2008 with the perinatal problems. So, Zografaki et al. [23] associated the fact that from 2008 to 2014 GDP per capita was reduced by 32% in Greece with the increase observed in the LBW rate in their country [23]. In the same way, Portugal [22] and Spain [5, 17] also have associated the financial crisis and its ensuing decrease in per capita GDP with the observed increment of LBW rates during that period. Our findings support this idea, given that we observe an association between some economic and health system conditionings and the LBW rate. In particular, from our data, per capita GDP was inversely associated with LBW, although a thousand dollar ($1000) increase in GDP only accounted for a 0.012% decrease in LBW, as already mentioned above.

Along this same line, Varea et al. [5] mentioned how the impact of the 2008 economic crisis introduced changes into the healthcare systems of some European countries, affecting per capita GDP spending on healthcare systems, among other factors [5]. This, in its turn, appears to have had a negative impact on pregnant women’s health and foetal development, as registered in some European countries such as Ireland or Greece. Varea et al. [5] therefore concluded that per capita GDP decrease is, by extension, a significant risk factor for LBW. Similarly, Sidebotham et al. [4] estimated that women who do not have adequate access to antenatal care can have problems in their own wellbeing and that of their foetus, which can result in LBW. Our results also support this idea, with the observed inverse relationship between health care system organization such as health coverage, public health system coverage or the number of hospitals per million inhabitants and LBW rate emanating from our data (Table 3).

Additionally, some authors did not consider the financial crisis as the sole responsible for the increase of LBW, but also the measures adopted by governments to deal with the crisis. As Rajmil et al. [24] pointed out, some governments decided to preserve the public systems while others chose to make cuts in health and other public services. Rajmil et al. [24] used the Cyclically Adjusted Primary Balance (CAPB), published by the International Monetary Fund (IMF [26]) as a measure of austerity concerning the decisions taken by the different governments. They found that countries with higher CAPB tend to have increase in the LBW percentages. Again, our results support this view, given that countries like Greece, Portugal, Spain, or UK (Fig. 1f and d, respectively) are categorized as countries implementing high austerity levels by Rajmil et al., and correspondingly, our data show a higher LBW rate for them too.

Our finding that the investment in private health care is directly associated with LBW could be explained by the exponential increase in assisted reproduction procedures, which are mainly in the hands of the private sector [27]. Rich regions tend to spend more on assisted reproductive techniques [28] . This fact is closely related with LBW, mainly as a result of multiple pregnancies and an increase in preterm birth, as reported by Goisis et al. [27]. They observed that 13% of children conceived by means of assisted reproduction showed LBW, as compared to 3% resulting from natural pregnancies. Also, women residing in rich regions tend to have their first pregnancy later than women belonging to poorer regions [29], and high age at first pregnancy is also intimately associated with LBW [6–8]. Besides the influence of the increased use of assisted reproduction procedures, other factors could explain this association, as Silva et al. (2006 and 2010) [30, 31] describe with the paradox of LBW. What this term refers is the fact that the LBW rates were lower in less developed areas in Brazil. As they concluded, there are several reasons to observe this result, such as the higher caesarean section rates or the improve medical care that gives the capacity of detecting some conditions encouraging professional to act, increasing the LBW rate. This paradox is also supported in our results given that higher rates of pediatricians and obstetricians showed association with higher rates of LBW, maybe to reduce the stillbirths.

Our analysis was restricted to developed countries included in the OECD; therefore, our results cannot be generalized to other countries. For instance, regarding the American continent, only four countries were considered, including two of the more developed countries in the world (the U.S. and Canada) and one of the most developed countries in Southern America. Accordingly, the trends we have described in Fig. 1a could well not apply to the rest of the continent. Similar limitations to the generalizability of our results could be noted regarding other continents, perhaps with the exception of Europe.

Our study has some limitations. Firstly, our analysis is ecological in nature, so causality cannot be invoked. Both collinearity and reverse causality could also be sensible explanations in some associations; for instance, higher LBW rates could be both the cause and the result of higher obstetrician ratios. Secondly, statistics were recorded at country level, allowing the use of different definitions between countries despite OECD standardization. In this regard, even definition changes within individual countries are possible throughout the studied period, leading to abrupt departures from the background trend (as shown for Mexico in Fig. 1a). Thirdly, some relevant factors such as information on gestational age, schooling or maternal parity, which have proximal relationship with LBW, have not been included in the analysis because those data were not available in the OECD data base. For the same reason, this study does not considerate the newborns weighing less than 2500 g as another category, which has an impact over perinatal and neonatal cares as well as on infant morbidity and mortality. Finally, country-level data, as used in this study, could mask within country differences in socioeconomic conditions, health system structure and LBW rates and trends.

## Conclusion

In OECD countries, LBW rates are related to contextual country characteristics such as GDP per capita, which is inversely related to LBW rate. Health care system factors, including health care coverage or investment in public health system, are directly associated with lower LBW rates.

## Supplementary Information

**Additional file 1: Table S1.** List of countries in the OECD and region assigned for the purpose of this paper.

## Data Availability

We gathered data for all 35 OECD countries (Supplementary Table 1) for the period ranging from 2000 to 2016 from the OECD data base (https://data.oecd.org/; last access: 19/October/2018).

## Abbreviations

LBW: Low birth weight
OECD: Organization for Economic Co-operation and Development
CI: Confidence interval
GDP: Gross Domestic Product
