# Supplementary material for: Low birth weight trends in Organisation for Economic Co-operation and Development countries, 2000–2015: economic, health system and demographic conditionings
Source: BMC Pregnancy Childbirth. 2021 Jan 6;21:13. doi: 10.1186/s12884-020-03484-9 (PMC7789240; doi:10.1186/s12884-020-03484-9)
Supplement: Supplementary file 1 — Additional file 1: Table S1. List of countries in the OECD and region assigned for the purpose of this paper. [file 12884_2020_3484_MOESM1_ESM.docx]

Supplementary Table 1. List of countries in the OECD and region assigned for the purpose of this paper

| **Country** | **Region** | **Country** | **Region** |
| --- | --- | --- | --- |
| Australia | Other region | Korea | Other region |
| Austria | Central Europe | Latvia | East Europe |
| Belgium | Central Europe | Luxembourg | Central Europe |
| Canada | America | Mexico | America |
| Chile | America | Netherlands | Central Europe |
| Czech Republic | East Europe | New Zealand | Other region |
| Denmark | North Europe | Norway | North Europe |
| Estonia | East Europe | Poland | East Europe |
| Finland | North Europe | Portugal | South Europe |
| France | South Europe | Slovak Republic | East Europe |
| Germany | Central Europe | Slovenia | East Europe |
| Greece | South Europe | Spain | South Europe |
| Hungary | East Europe | Sweden | North Europe |
| Iceland | North Europe | Switzerland | Central Europe |
| Ireland | North Europe | Turkey | Other region |
| Israel | Other region | United Kingdom | North Europe |
| Italy | South Europe | United States | America |
| Japan | Other region |  |  |
